# Updating dual-specificity tyrosine-phosphorylation-regulated kinase 2 (DYRK2): molecular basis, functions and role in diseases

**DOI:** 10.1007/s00018-020-03556-1

**Published:** 2020-05-27

**Authors:** Alejandro Correa-Sáez, Rafael Jiménez-Izquierdo, Martín Garrido-Rodríguez, Rosario Morrugares, Eduardo Muñoz, Marco A. Calzado

**Affiliations:** 1 Instituto Maimónides de Investigación Biomédica de Córdoba (IMIBIC), Avda. Menéndez Pidal s/n., 14004 Córdoba, Spain; 2 Departamento de Biología Celular, Fisiología E Inmunología, Universidad de Córdoba, Córdoba, Spain; 3 Hospital Universitario Reina Sofía, Córdoba, Spain

**Keywords:** DYRK2, Phosphorylation, Kinase, Cell cycle, Apoptosis, Disease, Cancer

## Abstract

Members of the dual-specificity tyrosine-regulated kinase (DYRKs) subfamily possess a distinctive capacity to phosphorylate tyrosine, serine, and threonine residues. Among the DYRK class II members, DYRK2 is considered a unique protein due to its role in disease. According to the post-transcriptional and post-translational modifications, DYRK2 expression greatly differs among human tissues. Regarding its mechanism of action, this kinase performs direct phosphorylation on its substrates or acts as a priming kinase, enabling subsequent substrate phosphorylation by GSK3β. Moreover, DYRK2 acts as a scaffold for the EDVP E3 ligase complex during the G2/M phase of cell cycle. DYRK2 functions such as cell survival, cell development, cell differentiation, proteasome regulation, and microtubules were studied in complete detail in this review. We have also gathered available information from different bioinformatic resources to show DYRK2 interactome, normal and tumoral tissue expression, and recurrent cancer mutations. Then, here we present an innovative approach to clarify DYRK2 functionality and importance. DYRK2 roles in diseases have been studied in detail, highlighting this kinase as a key protein in cancer development. First, DYRK2 regulation of c-Jun, c-Myc, Rpt3, TERT, and katanin p60 reveals the implication of this kinase in cell-cycle-mediated cancer development. Additionally, depletion of this kinase correlated with reduced apoptosis, with consequences on cancer patient response to chemotherapy. Other functions like cancer stem cell formation and epithelial–mesenchymal transition regulation are also controlled by DYRK2. Furthermore, the pharmacological modulation of this protein by different inhibitors (harmine, curcumine, LDN192960, and ID-8) has enabled to clarify DYRK2 functionality.

**Electronic supplementary material:**

The online version of this article (10.1007/s00018-020-03556-1) contains supplementary material, which is available to authorized users.

## Introduction

Dual-specificity tyrosine-phosphorylation-regulated kinase 2 (DYRK2) is an evolutionarily conserved enzyme belonging to the CMGC family of the eukaryotic kinome that encompasses cyclin-dependent kinases (CDK), mitogen-activated protein kinases (MAPK), glycogen synthase kinase (GSK), and CDK-like kinases (CLKs) [1]. The attribution DYRK (dual-specificity tyrosine (Y) phosphorylation-regulated kinase) highlights the peculiar biochemical properties of this protein kinase family, which possesses the ability to phosphorylate aromatic (tyrosine) as well as aliphatic (serine and threonine) amino acid residues. However, to acquire full catalytic activity, DYRKs require phosphorylation in a conserved “YxY” motif in their activation loop [2–6]. Regarding the homology of the kinase domain, DYRK family is classified into three subfamilies: pre-mRNA Processing Factor 4 kinases (PRP4s), homeodomain-interacting protein kinases (HIPKs), and DYRK kinases [1–3]. Within the DYRK subfamily phylogenetic analysis reveals three groups: Yak group (only in lower eukaryotes: YAK1p in *Saccharomyces cerevisiae* and YAKA in *Dictyostelium discoideum*), DYRK1, and DYRK2 groups. Specifically, DYRK1 group encompasses mammalian DYRK1A and DYRK1B, minibrain (Mnb) in *Drosophila melanogaster* and MBK1 *Caenorhabditis elegans*, whereas DYRK2 group contains several eukaryotic organisms, including mammalian DYRK2, *D. melanogaster* dDYRK2 (Smi35A) and dDYRK3, *C. elegans* MBK-2, and *Schizosaccharomyces pombe* POMP1p [1, 3, 7, 8]. In addition, the atypical class II DYRKs homolog TbDYRK has been described with a singular “FTY” activation loop motif, unlike DYRK kinases in other eukaryotes [9].

In mammals, DYRK subfamily is represented by two classes, class I (DYRK1A and DYRK1B) and class II (DYRK2, DYRK3 and DYRK4) [10]. Apart from the common central kinase domain, there are clear sequence differences between the subclasses defining characteristics involving subcellular localization, substrate specificity, and tissue distribution. Unlike class I DYRKs, which are mainly localized in the nucleus, class II DYRKs are predominantly localized in the cytoplasm. Class I and II subfamily members share a conserved central kinase domain and an adjacent N-terminal DH-box (DYRK homology box) but they possess different extended N- and C- terminal regions [1]. In this review, we intend to summarize in an ordered and detailed way the state of the art of DYRK2 kinase, making a deep review of the published literature as well as the available information in several databases.

## Sequence and structure

**Fig. 1:**
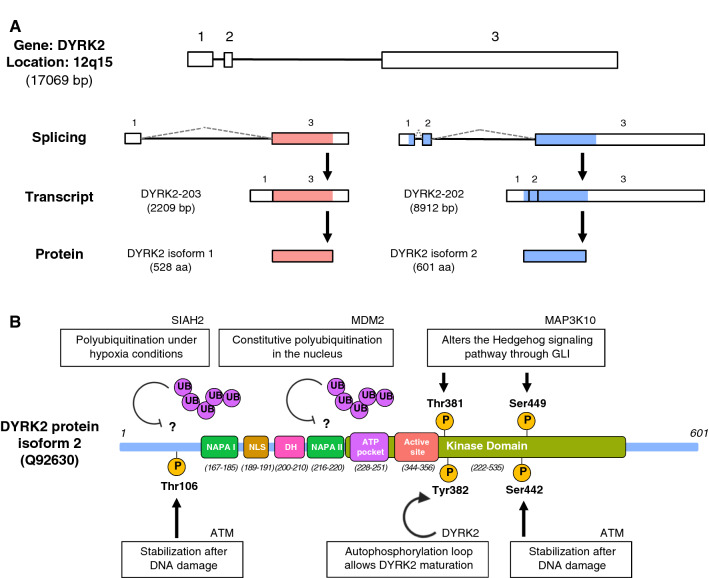
Scheme of DYRK2 genomics, transcripts, protein, and post-translational modifications. **a** Illustrative DYRK2 human expression processing. Exons are presented as rectangles and introns as lines and depending on the DYRK2 transcript, a specific splicing event is shown. Transcript and protein lengths as well as chromosomic location are indicated. Translated and untranslated exons are presented with color and white, respectively. DYRK-202 transcript includes exon 2, thus producing a larger transcript (8912 bp) and a protein of 601 aa (pink). On the other hand, DYRK2-203 transcript does not include this exon, thereby producing a shorter transcript (2209 bp) and a protein of 528 aa (blue). **b** Schematic representation of DYRK2 isoform 2 with described PTMs. Unique sequence motifs are presented in the illustration: two N-terminal autophosphorylation accessory regions (NAPA I and NAPA II), the nuclear localization signal (NLS), DYRK-homology box (DH), the ATP binding pocket, the kinase domain and the active site. DYRK2 is coordinately regulated by phosphorylation and ubiquitination, by upstream modulators altering DYRK2 function, stability, and subcellular localization. The affected residues by those PTMs are represented, as well as the autophosphorylation residue (Tyr382)

DYRK2 is widely considered to be the most important member among the class II DYRK protein subfamily [2–4, 11]. Database searches have identified different ESTs (expressed sequence tags) in the human genome, mapping this protein to chromosome 12q15 with a 17069 base pair length [12]. Moreover, DYRK2 orthologue genes are present in 99 mammal species, while 13 paralogue genes have been identified in other species. According to the Ensembl Genome Browser (January 2020), six different splice variants of DYRK2 have been identified [1], two of which have been isolated. DYRK2-203 transcript codifies 528 amino acids protein, being this sequence considered the most prevalent DYRK2 isoform or isoform 1 (NCBI Reference: NP_003574.1). DYRK2-202 produces a 601 aa sequence encoding a larger N terminus of 73 aa (DYRK2 isoform 2; NCBI Reference: NP_006473.2) (Fig. 1a). Although DYRK2 isoform 1 has been described as the most prevalent, the majority of databases as well as some articles use DYRK2 isoform 2 sequence. Consequently, throughout this review, DYRK2 information will be referred to isoform 2.

Many existing studies in the literature have examined in detail the structure and domains of this protein. In the last few years, much more information on DYRK2 structure based on X-ray crystallography has become available. In 2013, Soundararajan et al. presented DYRK2 protein structure (PDB: 3KL2) [4]. In a major advance in 2018, Banerjee et al. described DYRK2 crystal structure with curcumine (PDB: 5ZTN) [13] and 1 year later with LDN192960 (PDB: 6K0J) [14]. There have also been numerous studies to investigate the functional roles of DYRK2 domains. First, there are two common features shared among all DYRKs members. One is the presence of a distinctive motif called the DYRK-homology box (DH box), which is necessary for the formation of tertiary structure, and the other is the kinase domain (residues 222-535) [1]. Additionally, and like the other DYRK class II members, it presents two NAPA (N-terminal autophosphorylation accessory region) domains (NAPA1 and NAPA2), which provide a chaperone function, thus enabling DYRKs to catalyze self-phosphorylation in the activation loop tyrosine [3, 4, 6, 15, 16]. The presence of an “activation loop” segment (Tyr380-Thr-Tyr382) where phosphorylation of Tyr382 allows DYRK2 maturation is worth mentioning. Other residues of important relevance are the DYRK2 active site Asp348 and the ATP binding site Lys251. Ultimately, another DYRK2 feature shared with all DYRK members (except DYRK3) is the presence of a nuclear localization signal (NLS) (residues 189-191) enabling DYRK2 translocation into the nucleus (Schematic representation Fig. 1b) [14, 17].

## Expression, regulation, and post-translational modifications

**Fig. 2:**
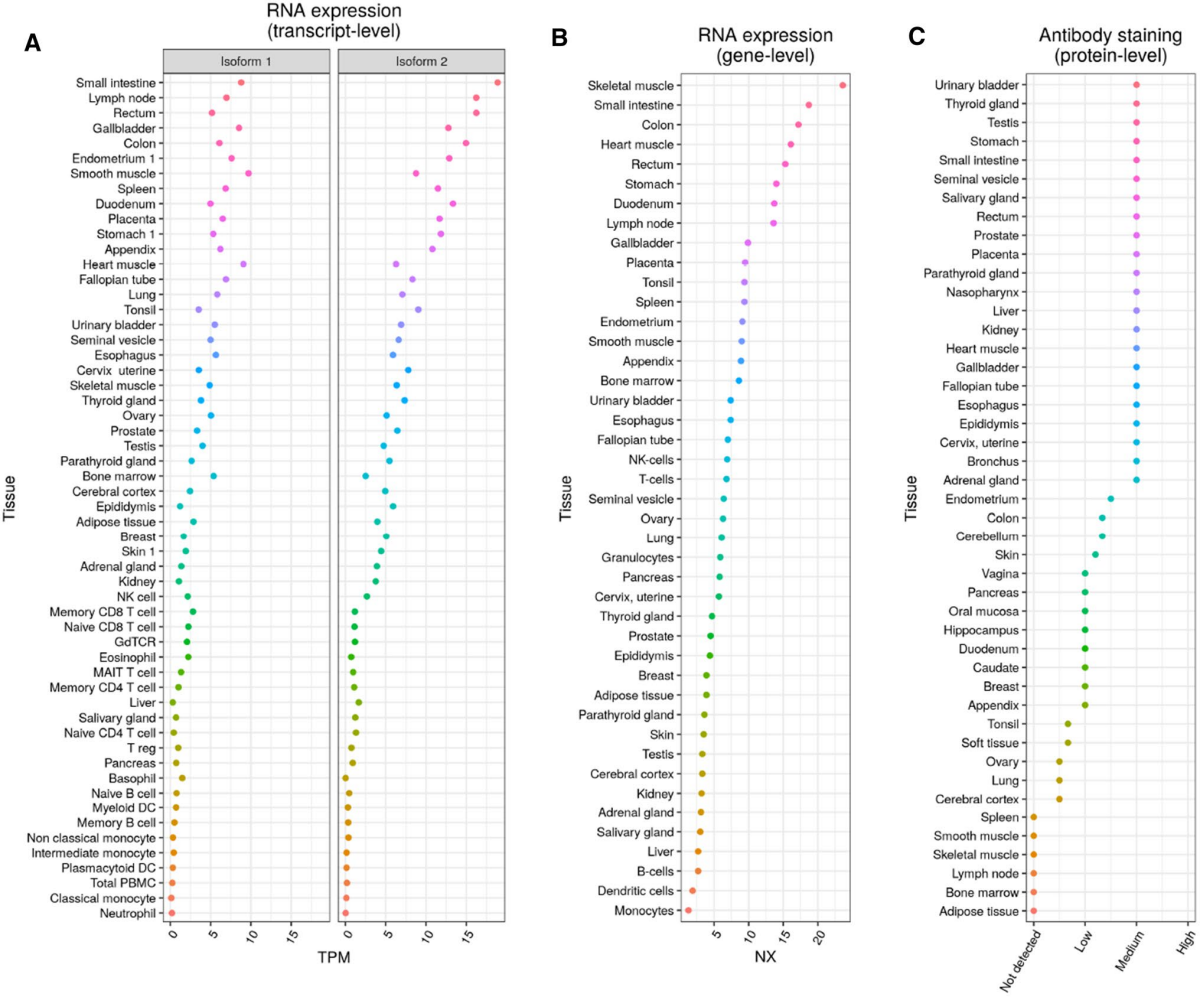
DYRK2 expression in normal tissue. **a** RNA expression at transcript level for DYRK2 isoforms 1 (short) and 2 (long). Points represent the averaged transcripts per million (TPMs) for each tissue (*y*-axis). **b** DYRK2 RNA expression at gene level as measured by the Human Protein Atlas combined score (NX), which integrates three sources of transcriptomic data (Human Protein Atlas, GTEx and FANTOM5). **c** DYRK2 protein expression measured through antibody staining. Points represent averaged staining by tissue, after discretizing data (Not detected, 0; Low, 1; Medium; 2, High; 3). Tissues were ordered from higher (up) to lower (down) expression, except for A, where the average expression of both isoforms was used. Data were obtained from the Human Protein Atlas database (https://www.proteinatlas.org/about/download) [103]

DYRK2 presents an expression pattern that varies across tissues and which depends on the measured biological level. On the one hand, tissues such as small intestine or heart muscle show a high expression of DYRK2 that correlates well from RNA isoform level to protein level. On the other hand, in lymph nodes or skeletal muscle, the high RNA expression does not correlate with the protein abundance detected through antibody staining, suggesting a tissue-dependent regulation of DYRK2 at protein level (Fig. 2).

A proper understanding of DYRK2 regulatory mechanisms would certainly help to clarify its role in diseases, especially in tumoral progression. At the transcriptional level, it has been described that KLF4 repressed *DYRK2* gene expression in leukemic stem/progenitor cells [18]. Similarly, recent evidence shows that DNMT1 downregulates *DYRK2* gene expression methylating its promoter, thereby increasing the proliferation of human colorectal cancer cells [19, 20]. In the same way, miR-662, miR-208a, miR-338-3p, miR-499, and miR-187-3p directly downregulate *DYRK2* expression in colorectal cancer cells, fibroblasts, human intestinal cancer (HIC) cells, cardiomyocytes, and liver cancer cells, respectively [21–25].

Like many other kinases, DYRK2 activation requires phosphorylation in an activation segment [26]. However, instead of requiring an upstream kinase, DYRKs catalyze self-phosphorylation to fully activate the kinase. In 2005, Lochhead et al. concluded that autophosphorylation of a crucial tyrosine residue in the activation loop is necessary for a correct kinase functionality. This event is mediated by a transitional intermediate version of DYRK resulting in a full kinase activation and a restricted kinase activity to Thr and Ser residues [3, 6]. Although DYRK self-phosphorylation has been considered as a one-time event, other groups have suggested that phosphorylation in its mature form could contribute to kinase fully activation [27]. In contrast with an increasing number of publications describing the presence of DYRKs active form autophosphorylation [4, 28–31], no information has yet been published addressing this event for DYRK2 (Fig. 1b).

In the case of post-translational modifications (PTMs), DYRK2 is tightly regulated in normal conditions by MDM2 ubiquitin ligase in the nucleus. Because of DNA damage, ATM kinase phosphorylates DYRK2 short isoform in Thr33 and Ser369 (Thr106 and Ser442 in DYRK2 long isoform). Thus enabling DYRK2 disassociation from MDM2 and consequent nuclear stabilization. This event finally leads to higher Ser46 phosphorylation of p53 [17, 32]. In addition, our group described that under hypoxic conditions, SIAH2 polyubiquitinates and degrades DYRK2. As a result, DYRK2-mediated Ser46 phosphorylation of p53 is compromised [33]. In a major advance, a high throughput genome-scale screening was performed in 2008, identifying that MAP3K10 directly phosphorylates DYRK2. These phosphorylations on DYRK2 short isoform, Thr308 and Ser376 (Thr381 and Ser449 in DYRK2 long isoform) alter its activity toward GLI, thus modifying the Hedgehog signaling pathway (Fig. 1b) [34]. A recent article on this topic showed that LPS induces DYRK2 nuclear translocation, thereby altering its protein levels [35]. Finally, it has been reported the Cep78 role inhibiting EDD-DYRK2-DDB1^VprBP^ complex, thus compromising CP110 ubiquitination and modifying centriole length and cilia assembly [36]. Apart from the mentioned PTMs directly derived from original works, additional DYRK2 modifications were collected from PhosphoSitePlus [37] and iPTMNet [38] and are included in Supplementary Table 1.

## Mechanisms of action

DYRK2 exerts its kinase function by phosphorylating Ser/Thr residues, and although at first it was considered a Pro-directed kinase, posterior studies showed that it affects substrates with a diversity of recognition motifs [4]. The presence of the arginine residue at − 2 position and proline at + 1 turns out to be essential, as reported by Campbell and Proud in 2002 by in vitro experiments [39]. Despite this, a certain degree of variability has been described, sometimes referring to the arginine residue at − 1 position [40]. Taken together, all these data result in a consensus sequence sensitive for DYRK2 phosphorylation: ‘Rx(x)S/TP’ [4]. Nevertheless, it is important to remark that this pattern is not necessarily present in all phosphorylation sites. All the substrates described to date are summarized in Table 1.

**Table 1:** DYRK2 substrates

Substrate	Phosphosites	Organism	GSK priming	Methodology	Reference
CARHSP1	Ser30, Ser32, Ser41	Human		In vitro assay; mass spectrometry	[110]
CRMP4	Ser522	Human	Yes	In vitro assay; point mutations	[111]
DCX	Ser306	Human		Point mutations	[112]
EIF2B5	Ser544	Human	Yes	In vitro assay; mass spectrometry; phospho-specific antibody; point mutations	[45]
	Ser539	Rat			
	Ser540	Mouse			
EIF4EBP1	Ser65, Ser101	Human		In vitro assay; point mutations; phospho-specific antibody	[113]
	Ser64	Rat			
GLI2	Ser388, Ser1011	Human		In vitro assay; mass spectrometry; phospho-specific antibody; point mutations	[34]
	Ser385, Ser1011	Mouse			
GYS1	Ser640	Human		In vitro assay; phospho-specific antibody; point mutations	[114]
	Ser640	Rabbit			
HSF1	Ser320, Ser326	Human		In vitro assay; mass spectrometry; phospho-specific antibody; point mutations	[64]
	Ser307, Thr323, Ser363	Human		In vitro assay; mass spectrometry	
H3F3A	Thr45	Human		In vitro assay; mass spectrometry	[115]
JUN	Ser243	Human	Yes	In vitro assay; phospho-specific antibody; point mutations	[44]
KATNA1	Ser42, Ser109, Thr133	Human		In vitro assay; phospho-specific antibody; point mutations	[51]
MYC	Ser62	Human	Yes	In vitro assay; phospho-specific antibody; point mutations	[44]
NDEL1	Ser336	Mouse	Yes	Mass spectrometry; phospho-specific antibody	[59]
NFAT1	?	Drosophila	Yes	Point mutations	[61]
NOTCH1	Thr2512	Human		In vitro assay; phospho-specific antibody; point mutations	[62]
TP53	Ser46	Human		In vitro assay; phospho-specific antibody; point mutations	[32]
RPT3	Thr25	Human		In vitro assay; phospho-specific antibody; point mutations	[66]
SIAH2	Ser16, Thr26, Ser28, Ser68, Thr119	Human		In vitro assay; mass spectrometry; phospho-specific antibody; point mutations	[33]
SNAIL	Ser104	Human	Yes	In vitro assay; phospho-specific antibody; point mutations	[43]
STAT3	Ser727	Human		In vitro assay; phospho-specific antibody	[116]
	Ser727	Mouse		In vitro assay; mass spectrometry	[117]
MAPT/Tau	Thr212	Human	Yes	In vitro assay; mass spectrometry; phospho-specific antibody; point mutations	[45]
TBK1	Ser527	Human		Phospho-specific antibody; point mutations	[63]
TERT	Ser457	Human		In vitro assay; phospho-specific antibody; point mutations	[52]

List of DYRK2 substrates indicating phosphosites, GSK priming function and methodology

Among its phosphorylation activities, DYRK2 has been referred as a priming kinase for GSK3β [41–46]. Glycogen synthase kinase 3 beta (GSK3β) is a Ser/Thr kinase with a wide range of substrates and functions [47, 48]. Although it is not essential, GSK3β usually requires the previous phosphorylation of the substrate to properly exert its activity [49, 50]. In this sense, DYRK2 phosphorylates some substrates that are subsequently recognized by GSK3β, such as CRMP4, c-Myc, c-Jun, SNAIL, or eIF2Bε [43–46] (Table 1). In all those cases, the priming phosphorylation by DYRK2 is followed by a second phosphorylation by GSK3β, leading to the ubiquitination of these substrates and their consequent proteasomal degradation.

Besides the kinase activity, it has been described that DYRK2 also acts as a scaffold protein. In 2009, Maddika et al. described that DYRK2 functions as an intermediate adaptor protein between EDD and DDB1 components of the EDVP (EDD-DDB1-VprBP) E3 ligase complex, especially in the G2/M cell cycle phase. Additionally, they showed that DYRK2 also phosphorylates katanin p60, one of its substrates, leading to its ubiquitination and subsequent proteasomal degradation [51]. A similar action was then reported for TERT (telomerase reverse transcriptase). This enzyme is another of the EDVP substrates which has and presents an essential role in telomere maintenance, thus supporting genomic stability [52]. More recently, it has been also reported that DYRK2-dependent phosphorylation of CP110 (Centrosomal Protein of 110 kDa) leads to DYRK2-EDVP E3 ligase complex recognition of the substrate and its consequent proteasomal degradation. The same study reveals a negative regulator of this CP110 degradation event: Cep78 (Centrosomal protein of 78 kDa) [36].

## Biological functions

**Fig. 3:**
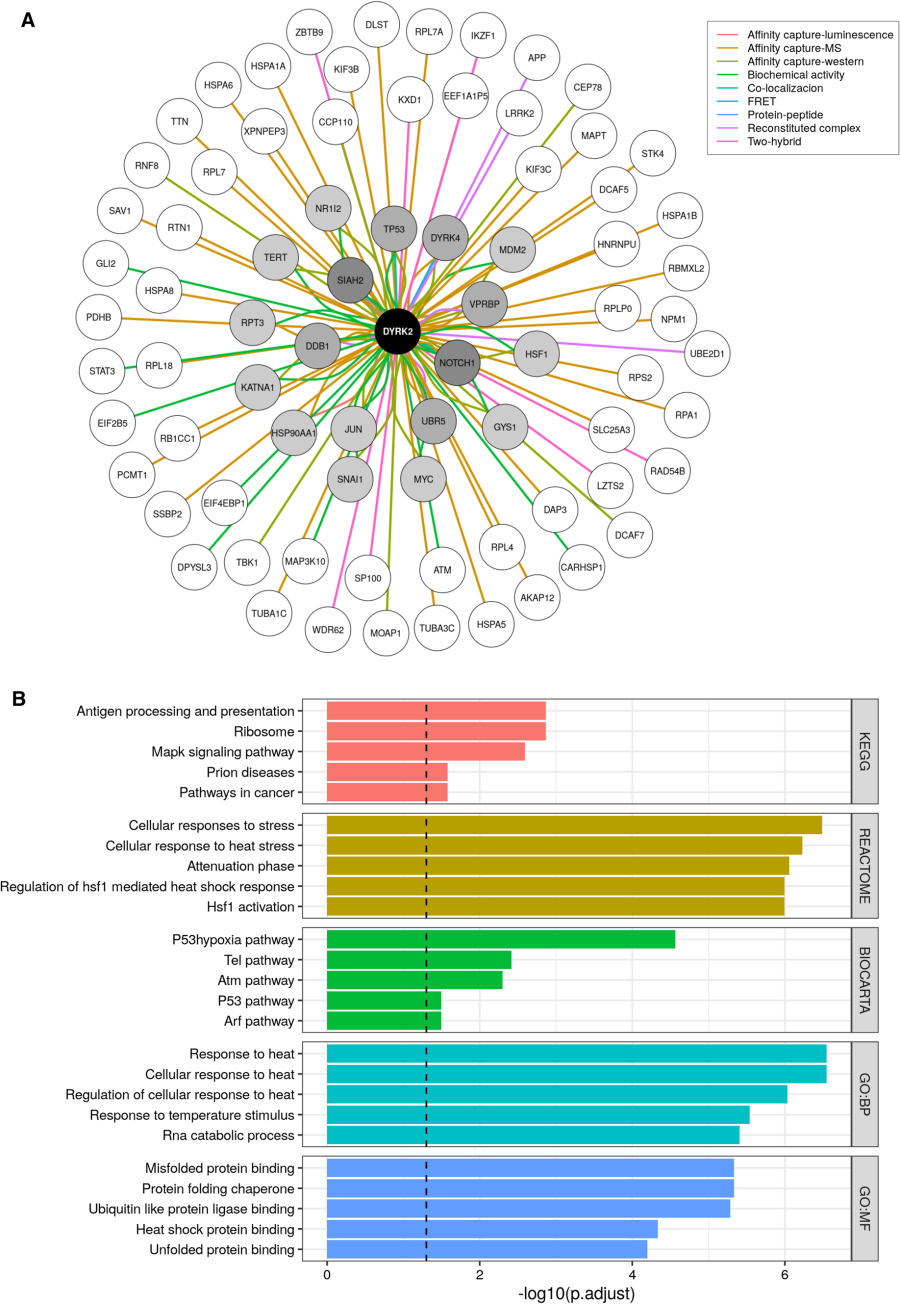
DYRK2 interactome. **a** A list of physical interactors for DYRK2 was obtained from BioGrid (https://downloads.thebiogrid.org/BioGRID/) [104] and completed with our list of literature-curated DYRK2 interactors. On the network, the edge color indicates the experimental system that supports an interaction, while the node color represents the number of different experimental systems supporting a given interactor (white, 1 method; dark grey, 4 methods). **b** Pathway and gene ontology over-representation analysis results for the 78 genes comprising our DYRK2 interactome. Functional categories were obtained from MSigDb (https://www.gsea-msigdb.org/gsea/msigdb/index.jsp) [105] and the over-representation analysis was carried out with clusterProfiler [106]. The bar plot represents the significance for the top five over-represented pathways or terms by functional category as the -log10 transformed adjusted *P* value. Dashed line indicates an adjusted *P* value cutoff of 0.05

One way to predict the functionality of a protein kinase is evaluating its interactome in detail. For this reason, we have reconstructed the DYRK2 interactome using a combination of the physical interactors from the BioGrid database and our list of literature-curated interactors (Fig. 3a). The posterior functional characterization of these data through an over-representation analysis has allowed us to have a global vision of the contribution of DYRK2 to different cell functions, amongst which we can mention “Pathways in cancer”, “Cellular response to stress”, or “ATM pathway” (Fig. 3b). Furthermore, we have performed a detailed study of the available literature to date, where DYRK2 role stands out in:

Cell survival: Among the different pathways controlling cell-damage responses, p53 is certainly the most important protein inducing apoptosis-related genes after kinase phosphorylation [53, 54]. In this sense, Taira et al. in 2007 identified DYRK2 as a novel kinase that phosphorylates p53 in Ser46, inducing apoptosis after DNA damage [32]. This work undoubtedly centred the attention of the scientific community in studying DYRK2 role in the apoptosis response. Subsequent articles showed, for example, the ability of ATM to phosphorylate DYRK2 at Thr33 and Ser369 and enable its stabilization after DNA damage. Consequently, this event controls p53-dependent DYRK2 apoptotic response [17]. In 2012, our group proved that under hypoxic conditions DYRK2 expression is compromised due to the ubiquitin ligase SIAH2 activity. Therefore, this event reduces p53 activation and increases chemotherapy resistance [33]. More recently, KLF4 inhibition of DYRK2 has proven to decrease p53 activation promoting leukemic stem/progenitor cells survival and self-renewal [18].

On the other hand, despite these findings of the DYRK2 role as a proapoptotic kinase, other functions of this kinase controlling DNA stability have emerged. That is the case of the DNA double-strand break (DSB) repair process. After genotoxic stress, DYRK2 interacts and colocalizes with RNF8 regulating its ubiquitination activity. Intriguingly, silencing DYRK2 suppresses the mono-ubiquitination of γ-H2AX and the foci formation of the p53-binding protein 1 (53BP1), thus failing to repair the DNA damage. This evidence suggests that both proteins cooperate in DSB repair [55].

Cell development and differentiation: Accumulating studies have revealed that DYRK2 has important roles in cell development and differentiation in several species. Regarding *D. melanogaster*, it has been described that the orthologue DmDyrk2 is critical for nervous system development. Specifically, DmDyrk2 expression is triggered in late larva state when the eye is patterned in the third antennal segment. This structure is responsible for smell and proper visual system development [56]. In the light of these observations, some authors have suggested a possible cross talk between DmDYRK2 and the Hedgehog signaling pathway [40]. In the case of *C. elegans,* several studies have found that the DYRK2 orthologous gene MBK-2 is essential for embryo viability. On the one hand, MBK-2 plays an important role in cytokinesis in the early embryo due to its regulation of maternal protein degradation [57]. Similarly, OMA-1 and OMA-2 MBK-2-mediated phosphorylation is necessary to convert this protein into key regulators of 1-cell embryo development [42]. In Zebrafish *(Danio rerio)*, DYRK2/CDK5 phosphorylation of Dpysl2 and Dpysl3 (Dihydropyrimidinase-related protein 2 and 3) contributes to properly position Rohon–Beard neurons and neural crest cells in neural tube formation [41]. Additionally, there is a positive correlation between DYRK2 and MyoD (myoblast determination protein 1) during the early stages of embryo development, suggesting that fast-twitch muscle differentiation depends on this regulation [58]. Similarly, in mammals, DYRK2 emerged as responsible for sequential phosphorylation of NDEL1 modulating F-actin dynamics, thus altering both axonal and dendritic neurites development [59]. Moreover, it has been proposed that this event leads to fibroblast proliferation and myofibroblast differentiation via NFAT phosphorylation [23]. Finally, a recent article found an orthologue DYRK2 gene in *Trypanosoma brucei* (TbDYRK2) with a transcriptional function in the regulation of this parasite developmental pathway [60].

Gene transcription: DYRK protein kinase family has been considered a master regulator of gene expression programs [3]. In 2006, DYRK1A and DYRK2 were first identified as novels regulators of the transcription factor NFAT. DYRK2 phosphorylates the conserved serine-proline repeat 3 (SP-3) motif of the NFAT regulatory domain priming further phosphorylation by GSK3β and CK1 [61]. As discussed above, DYRK2 phosphorylation of p53 in Ser46 triggers the activation of the apoptosis-related pathway. Besides that, TUNEL assays physiologically suggested an increase of the apoptosis, those effects were also visualized in the *p53AIP1* expression increase [32]. Next, Varjosalo et al. demonstrated that DYRK2 phosphorylates Gli2 and inhibits Sonic hedgehog (Shh) signaling, thereby resulting in a GLI protein expression reduction [34]. Similarly, it has been described as a priming kinase of c-Jun and c-Myc [44]. Very recently, our group revealed the DYRK2 implication in NOTCH1 signaling pathway mediating NOTCH1-IC degradation. NOTCH1-IC regulation mediated by DYRK2 reveals an increase not only in NOTCH1 downstream genes, such as *Hes1* and *Hes5*, but also in cell migration and invasiveness [62]. Additionally, DYRK2 negative regulation of TBK1 has shown to take part in the IFN-β-mediated cellular antiviral response [63]. DYRK2 has also been reported to negatively regulate the proteotoxic-stress response pathway regulating Heat Shock Factor 1 (HSF1) [64]. In 2007, Imawari et al. reported DYRK2 regulation of CDK14 through the Wnt/β-catenin signaling pathway [65].

Proteasomal regulation: The 26S proteasome is a crucial protein complex in eukaryotes responsible for degrading cellular proteins, with enormous importance in cell cycle regulation and the development and progression of numerous cancers. In 2016, Guo et al. revealed the DYRK2 implications in 26S proteasome complex as the primary Rpt3 Thr25 phosphorylation kinase controlling G1/S transition. DYRK2 inactivation and consequent loss of phosphorylation are enough to reduce protein activity, slowdown cell proliferation, and potentiate bortezomib anti-growth effect [13, 14, 66].

Microtubule formation: As we pointed out before in this review, DYRK2 is part of the EDD–DDB1–VPRBP (EDVP) E3 ligase complex. This complex has been described to degrade katanin p60 due to the main action of DYRK2 by recognizing and phosphorylating katanin p60, thus controlling mitotic transition. Silencing either EDD or DYRK2 leads to defective mitotic progression [51]. Likewise, EDVP–DYRK2 complex controls the centrosome homeostasis (considered the major microtubule-organizing center in most eukaryotic cells) through a regulation mechanism mediated by Cep78 protein [36].

Other functions: Finally, to conclude this section, the literature has identified other areas where DYRK2 function is involved. Using a kinome-wide siRNA screen, DYRK2 was reported to negatively regulate the human pregnane X receptor (hPXR). Moreover, this observation was further validated by in vitro kinase and ubiquitination studies. It has been previously described that hPXR is part of the human liver detoxification system. Therefore, DYRK2 regulation of this system is a plausible idea [67]. On the other hand, DYRK2 negative regulation of eIF2Be in cardiac myocytes results in a reduced cardiomyocyte cell growth playing a physiological role in cardiomyocyte hypertrophy [68].

## DYRK2 and disease

Due to the wide range of biological functions of this protein, DYRK2 role in the development of human diseases is an increasingly important area. Although during the last 2 decades, an extensive work in this sense has been developed [35, 65, 69, 70], there are still remarkable controversies.

**Fig. 4:**
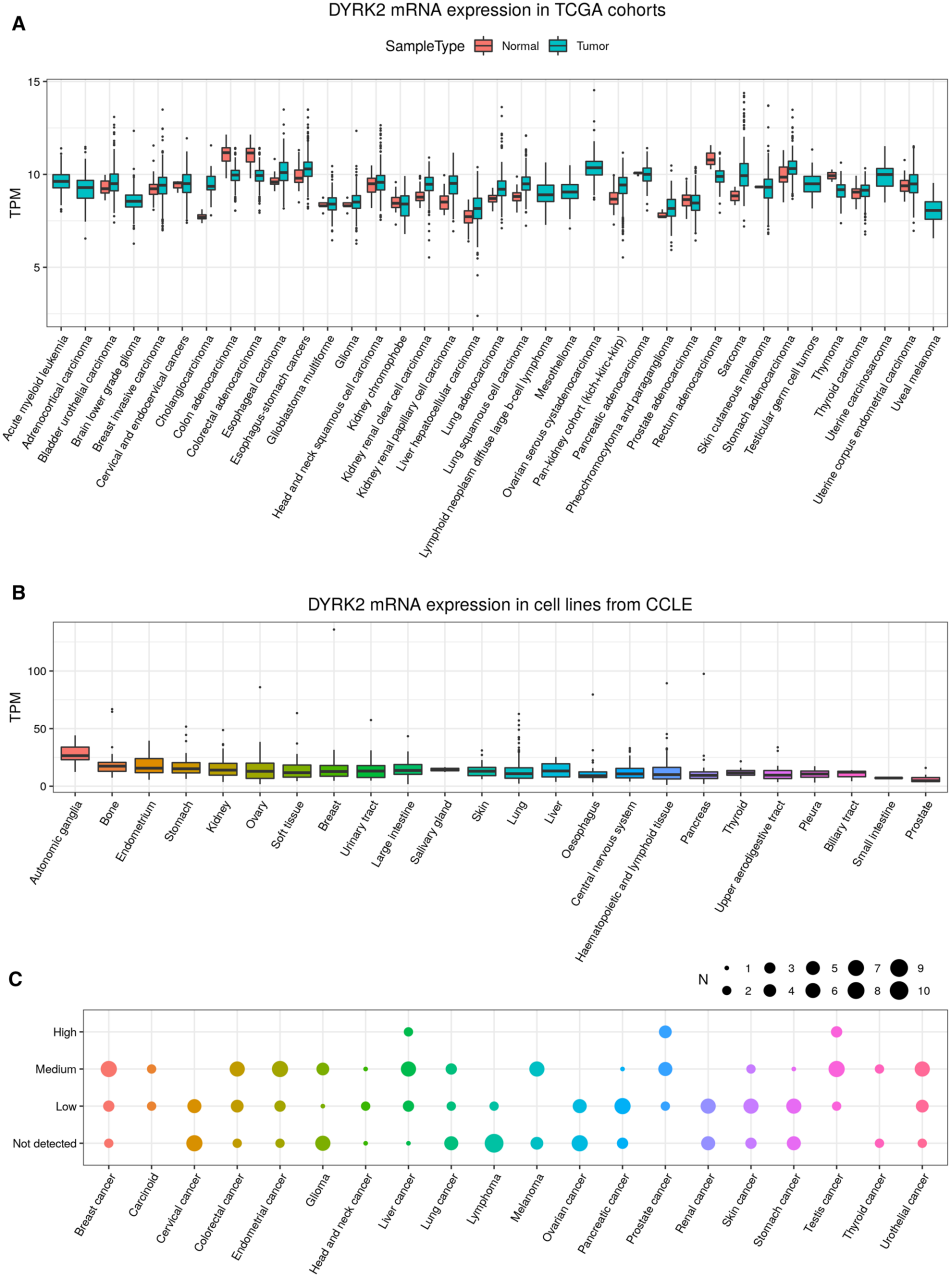
DYRK2 expression in cancer. **a** DYRK2 RNA expression across TCGA cohorts. Data were obtained with FireBrowseR [107]. The boxplot represents the DYRK2 transcripts per million (TPM) distributions for the tumor tissues of each cohort and when available, for matching normal tissues. **b** DYRK2 RNA expression on cell lines from the Cancer Cell Line Encyclopedia (https://portals.broadinstitute.org/ccle) [108] grouped by cell line origin (*X*-axis). **c** DYRK2 protein staining levels from the Human Protein Atlas pathology data (https://www.proteinatlas.org/about/download) [103]. For each cancer type (*X*-axis), the point size indicates the number of samples with a particular antibody staining level (*Y*-axis)

**Cancer:** DYRK2 expression in cancer may vary widely depending on the tissue as well as on the expression at the RNA or protein level. Likewise, there is a large difference of expression among the cell lines normally used in cancer research. Data collected from different resources are summarized in Fig. 4. In the same way, the bibliography includes many works which analyze DYRK2 expression in human tumor tissue compared to adjacent healthy tissue at different levels (Table 2). Although in most studies, a decrease in the expression is described (both at protein and RNA level) in tumor tissues compared to normal tissue, in some cases, such as ovarian cancer, it shows the opposite pattern. Also, nowadays there are certain pathologies where the available studies are not yet conclusive. This happens especially in breast, lung, and esophageal cancer, where more complete and detailed studies should be performed to clarify these differences. Likewise, and although to date, different DYRK2 mutations in tumors have been described (Fig. S1), additional studies are needed to evaluate its incidence and effects.

**Table 2:** Expression of DYRK2 in human tumor tissue compared with normal

Cancer type	Expression	Analyzed level	Role /function	References
Colorectal cancer	Low	RNA/Protein	Tumor suppressor	[19]
	Low	RNA/Protein	Tumor suppressor	[22]
	Low	Protein	Tumor suppressor	[44]
	Low	RNA/Protein	Tumor suppressor	[77]
Breast cancer	Low	Protein	Tumor suppressor	[43]
	Low	Protein	Tumor suppressor	[44]
*Triple-negative breast cancer*	High	RNA/Protein	Oncogenic	[14]
Hepatocellular carcinoma	Low	Protein	Tumor suppressor	[118]
	Low	Protein	Tumor suppressor	[80]
Lung cancer	High	RNA/Protein	Oncogenic	[69]
	Low	Protein	Tumor suppressor	[119]
Esophageal cancer	Low	Protein	Tumor suppressor	[44]
	High	RNA/Protein	Oncogenic	[69]
Chronic myeloid leukemia	Low	RNA	Tumor suppressor	[18]
Ovarian cancer	High	Protein	Oncogenic	[73]
Glioma	Low	Protein	Tumor suppressor	[70]
Kidney cancer	Low	Protein	Tumor suppressor	[44]
Anus cancer	Low	Protein	Tumor suppressor	[44]
Prostate cancer	Low	Protein	Tumor suppressor	[44]

The table lists the different studies where DYRK2 levels in tumor have been analyzed as compared to healthy tissue. We show the expression level, the analyzed level (protein or RNA) as well as the proposed function in carcinogenesis

As described above, DYRK2 modulates functions as important in cancer development as apoptosis, cell proliferation, or cell growth, among others. For that reason, it is not surprising that most of the DYRK2 knowledge is related to this pathology. Below we describe the DYRK2 role on the control of these functions.

Cell cycle: First, DYRK2 ability to affect G2 phase through the degradation of essential phase transition proteins was described. As discussed above, katanin p60 and TERT are phosphorylated and degraded by the EDVP–DYRK2 complex at G2/M transition [51, 52]. Later in 2012, Taira et al. described DYRK2 ability to degrade c-Jun and c-Myc, two transcription factors essential in the cell cycle control, by sequential phosphorylation together with GSK3β. They observed higher levels of the unphosphorylated forms after DYRK2 downregulation, followed by a shortened G1 phase and higher levels of breast cancer invasiveness [44]. The authors support that G1 phase prolongation leads to a delay in the expression of other key proteins such as Cyclin E [71]. Tumor growth was potentiated after DYRK2 silencing in xenograft models, thereby revealing the clear implications of this event in cancer development. In line with these results and supporting the importance of DYRK2 phosphorylation in G1/S transition, the ability to regulate 26S proteasome complex through Rpt3 Thr25 phosphorylation was described [66]. In this case and contrary to the previous evidence, loss of DYRK2 attenuated cell growth. Moreover, the same group later described how the inhibition of DYRK2 activity mediated by curcumin and LDN192960 reduces cell proliferation and induces cell death, thus compromising triple-negative breast cancer (TNBC) [13] and multiple myeloma (MM) growth [14]. These differences between both observations need to be clarified in the future and could be explained because of the use of different cell lines and the studied cancer subtype.

Apoptosis: As mentioned above, among its several functions, it has been reported that this kinase is a regulator of the apoptotic response. Different works sustain this role and the first of them showed that DYRK2 regulates p53-dependent apoptosis in response to DNA damage [32, 44]. This, together with the findings that DYRK2 is stabilized in the apoptotic response to DNA damage as a result of ATM-dependent Thr33 and Ser369 phosphorylation, reinforces the idea of DYRK2 as a proapoptotic protein. Other works are in line with this idea, for example, a recently published study showed that KLF4 protein inhibits DYRK2, reducing apoptosis and inducing survival of leukemic and stem progenitor cells [18]. Additionally, it has been demonstrated that DYRK2 suppression by knockout or inhibition with curcumin or LDN192960 leads to an increase of apoptosis in triple-negative breast cancer cells and xenografts due to a reduction of the 26S proteasome activity [13, 14]. Besides this, our group recently described that DYRK2 induces cell apoptosis in a NOTCH1-IC-dependent way using breast cancer cell lines [62].

On the other hand, several research works support an antiapoptotic function of DYRK2. In 2017, Yamamoto et al. described that under DNA damage conditions that promote double-strand break (DSB), DYRK2 recruits RNF8 collaborating to DNA repair [55]. It has also been reported that DYRK2 regulates proteotoxic resistance via HSF1, and that DYRK2 KO breast cancer cell lines show a higher apoptotic rate comparing to the wild type [64]. Finally, Wang et al. show that miR3883-3p targets DYRK2 in human intestinal cancer cells (HICs) after X-ray radiation, leading to an increase of the apoptosis of these cells [24].

EMT and metastasis: Another key event that takes place in cancer development and aggressiveness is the ability of the cells to migrate and invade different niches. In connection with this, it is remarkable to mention the role of DYRK2 in the control of epithelial to mesenchymal transition (EMT) and cell motility. In this sense, it has been described that DYRK2 is able to phosphorylate SNAIL as a priming kinase for GSK3β, inducing its degradation with the subsequent suppression of the EMT [72]. This DYRK2-dependent process leads to a reduction of the metastatic potential in human breast and ovarian cancer cell lines and xenografts [43, 73, 74]. In a recent study, Ryu et al. demonstrated that SNAIL phosphorylation can be avoided by the action of p38, then promoting EMT and metastasis in xenograft models of ovarian cancer [73]. In addition, DYRK2 has been reported to be implicated in the suppression of EMT in glioma since it is associated with decreased expression of Vimentin and SNAIL and stabilization of E-cadherin levels [70]. Additionally, in 2013, Mrugala et al. showed that DYRK2 protein levels are significantly reduced in high-grade glioma tissue compared to low-grade glioma tissue, suggesting its relevance in metastatic process and invasiveness potential [75]. In the case of colorectal cancer (CRC), it has been shown that DYRK2 compromises metastasis in CRC cell lines and xenografts and this also correlates with a better survival rate [76, 77].

Together with its role in EMT, DYRK2 also regulates several substrates related to cell motility and metastatic potential. NOTCH1 protein levels are increased in DYRK2 knockout human breast cancer cell lines, leading to a rise of the invasion and migration potential of these cells compared to the wild type [62]. Using DYRK2-depleted breast cancer cell lines, it has been described that this kinase mediates the invasion potential of these cells via CDK4 regulation [65]. Additionally, miR-622 has been reported to be a regulator of DYRK2 expression of CRC cells, modulating their migration and invasion, thus reinforcing potential DYRK2 antitumoral role [22]. Nevertheless, not all works state that DYRK2 negatively regulates invasion and metastatic potential of cancer cells. For example, it has been reported that DYRK2 inhibition by LDN192960 affects the tumorigenic potential of triple-negative breast cancer cells due to DYRK2 activity on 26S proteasome [13, 14].

Cancer stem cell (CSC) formation: To date there are several studies relating DYRK2 expression with this relevant cell population. First, Mimoto et al. proved that DYRK2 negatively regulates breast cancer stemness formation targeting KLF4 expression. In this work, the authors observed that DYRK2 inversely correlates with CD44^+^/CD24^−^ subpopulation and KLF4 expression. Consequently, DYRK2 downregulation leads to a sphere-forming ability, thus indicating self-renewal capacity [78, 79]. In the same sense and as we have previously mentioned, several studies have described DYRK2 ability to regulate c-Myc and SNAIL levels, both considered key stemness proteins [44]. Likewise, further efforts have been done to elucidate DYRK2 role in chronic myeloid leukemia (CML) stem/progenitor cells. In 2019, Park et al. demonstrated that SIAH2 ubiquitin E3 ligase inhibition using vitamin K3 produces the stabilization of DYRK2, which inhibits self-renewal via c-Myc depletion and p53 activation [18].

Cell survival: Studies conducted in different cancer patients show that DYRK2 correlates with the survival rate according to the specific cancer subtype. For example, there is a positive correlation of DYRK2 expression with patient survival in hepatocellular, non-small cell lung, pulmonary adenocarcinoma, colorectal and bladder cancers, and non-Hodgkin’s lymphoma [76, 77, 80–84]. On the contrary, other results have revealed that DYRK2 expression in neuroblastoma inversely correlates with patient survival [85]. In contrast to the previously observed positive relation in breast cancer [43, 86], Guo et al. observed an inverse correlation between DYRK2 expression and survival in the same cancer subtype [66]. Additionally, DYRK2 depleted and parental multiple myeloma cell lines such as MPC11 and 5TGM1 have been used in xenograft models, proving a negative relation with mice survival [14]. In the same line, this pro-survival function is related to higher chemotherapy response. It has been revealed that higher DYRK2 expression tumors are more susceptible to being treated using chemotherapy. Some well-known examples are bladder cancer, hepatocellular cancer, breast cancer, and non-small cell lung cancer [43, 80, 81, 83].

**Other diseases**: Together with the efforts to elucidate the regulatory mechanisms and implications of DYRK2 in cancer, the role of this kinase in other pathologies has been studied. For example, DYRK2 has been described as an essential kinase for virus-triggered IFN-β negative induction and the cellular antiviral response [63]. Similarly, in HIV-1 infection, DYRK2 orchestrates its effects on the regulation of microtubule dynamics during the virus replication cycle. HIV-1 accessory viral protein R (Vpr) possesses an important role moving to the centrosome through DCAF1 and subsequently constituting a complex with the ubiquitin ligase EDD-DYRK2-DDB1^DCAF1^ and Cep78. Consequently, HIV-1 Vpr hijacks EDD-DYRK2-DDB1^DCAF1^ complex to interfere with centrosome homeostasis contributing to HIV-1 pathogenesis [36]. Additionally, DYRK2 has been studied in the cardiomyocyte growth in the context of left ventricular hypertrophy (LVH). In this sense, DYRK2, as priming kinase of GSK3β, appears to be a potent negative regulator of the eIF2B activity and in cardiac hypertrophy [68]. In neuroinflammation diseases, DYRK2 has recently emerged as a potential molecular target to improve neuronal apoptosis. Specifically, DYRK2 takes part in the regulation of LPS-induced neuronal apoptosis, thus affecting the phosphorylating of NF-κB subunit p65, Akt, and p38MAPK [87].

## Pharmacological inhibition of DYRK2

To date, different molecules have demonstrated their ability to inhibit DYRK2 activity. Although the majority shows an effect on other members of the DYRK family or even on other kinases, some of these chemicals have shown enough specificity to help to elucidate DYRK2 functionality both in vitro and in vivo.

Harmine (7-methoxy-1-methyl-9H-pyrido[3,4-b]indole): Tricyclic β-carboline alkaloid isolated from *Peganum harmala L.,* this molecule is a high-affinity inhibitor of monoamine oxidase A (MAO-A) [88, 89] and probably the most widely used to study the chemical inhibition of DYRK family. Able to inhibit all the DYRK family, it is an ATP-competitive inhibitor with a potent activity against DYRK1A with an IC_50_ of 0.08 mM. Regarding DYRK2, it shows an IC_50_ of 0.8 mM [90, 91]. As a cell permeable inhibitor, it is commonly used to study DYRK family functionality [92]. It inhibits cell proliferation, migration, and invasion in several human cancer cell lines [85, 93].

Curcumin (diferuloylmethane): Active component of the perennial herb *Curcuma longa*, it presents multiple biological activities through different mechanisms of actions [94]. It occupies the ATP-binding pocket of DYRK2, inhibiting its activity in vitro with an IC_50_ of 5 nM, as well as pThr25 RPT3 phosphorylation in HEK293T cells stably transfected with Flag-DYRK2 in a dose-dependent manner, with maximum effects at 3–10 μM concentrations. It shows the ability to inhibit other members of the DYRK family, but to a lesser extent (DYRK1A, IC_50_: 190 nM; DYRK3, IC_50_: 20 nM). It has shown its ability to sensitise triple-negative breast cancer and multiple myeloma TNBC cell lines through the partial inhibition of the proteasome activity [13].

LDN192960 (3-(2,7-dimethoxyacridin-9-yl)sulfanylpropan-1-amine;dihydrochloride): Initially developed as an inhibitor of Haspin [95, 96], it occupies the ATP-binding pocket of DYRK2, inhibiting its activity in vitro with an IC_50_ of 13 nM, as well as Thr25 RPT3 phosphorylation in HEK-293T cells stably transfected with Flag-DYRK2 in a dose-dependent manner, with maximum effects at concentrations between 1–10 μM. It also inhibits other DYRK family members (DYRK1A, IC_50_: 122 nM; DYRK3, IC_50_: < 3 nM). Like curcumin, different approximations have demonstrated its ability to inhibit neoplastic progression in triple-negative breast cancer and multiple myeloma cell lines [14].

ID-8 (1-(4-Methoxyphenyl)-2-methyl-3-nitro-1H-indol-6-o): Compound with the ability to allow expansion of undifferentiated mouse embryonic stem cells [97], which interaction with DYRK2 was identified through affinity chromatography. Combined with Wnt, it enhanced hESC replating efficiency and displayed undifferentiated morphology [98].

7BIO (7-bromoindirubin-3′-oxime): A caspase-independent nonapoptotic cell death inducer derived from indirubin [99]. It acts as an ATP-competitive inhibitor on DYRK2 with an IC_50_ of 1.3 μM. It also inhibits DYRK1A with an IC_50_ of 1.9 μM [100]. Moreover, it is a potent inhibitor of FLT3, Aurora kinases B and C [99, 101]. It acts as an ATP-competitive inhibitor on DYRK2 with an IC_50_ of 1.3 μM. It also inhibits DYRK1A with an IC_50_ of 1.9 μM [100, 101].

AZ191 (N-[2-methoxy-4-(4-methylpiperazin-1-yl) phenyl]-4-(1-methylpyrrolo[2,3-c]pyridin-3-yl)pyrimidin- 2-amine): Selective ATP competitive that inhibits DYRK1B serine/threonine kinase activity without blocking autophosphorylation of the activation loop tyrosine residue. It inhibits DYRK2 in a similar way, with an IC_50_ of 1.89 μM. A reversible and cell permeable inhibitor, it can inhibit the phosphorylation of the DYRK1B substrate CCND1 and the levels of the cell-cycle regulators p21Cip1 and p27Kip1 in cells [102].

## Conclusions and perspectives

Although, during the last 20 years, new insights have been provided into DYRK2 biological function and its role in some diseases such as cancer, there is still little work on this kinase despite the relevance of some of its substrates. Its key role in the control of cell processes as important as those described in this revision shows the need of enlarging the knowledge of many biological, functional, and regulating aspects of this protein. On the one hand, it is remarkable that the small number of regulating mechanisms and stimuli able to modify the expression and activity of this kinase that we know to date. In this sense, an effort should be made to describe new post-translational modifications able to alter DYRK2 functionality, since the number and types described so far are surprisingly low. Similarly, new DYRK2 substrates should be described, thus opening the way to the discovery of new functions. These two aspects would no doubt help to clarify its role in the control of the cell cycle and apoptosis, which have not been clarified yet. On the other hand, it would be relevant to clarify the role of this kinase in tumor control and development. Even though the available literature to date mainly describes it as a tumor suppressor, this could not be the case in all tumor subtypes. This needs to be explained, above all in breast and lung cancer. Finally, and despite the existence of several molecules able to inhibit DYRK2 activity, it would be a challenge to research new and more specific molecules, which may allow us to pharmacologically modify the relevant functionality of this kinase in those required diseases.

### Electronic supplementary material

Below is the link to the electronic supplementary material.

Supplementary file1 (PDF 613 kb)

Supplementary file2 (PDF 97 kb)

## References

[1] Becker W, Joost HG (1998). Structural and functional characteristics of Dyrk, a novel subfamily of protein kinases with dual specificity. Progr Nucleic Acid Res Mol Biol.

[2] Soppa U, Becker W (2015). DYRK protein kinases. Curr Biol.

[3] Aranda S, Laguna A, de la Luna S (2011). DYRK family of protein kinases: evolutionary relationships, biochemical properties, and functional roles. FASEB J.

[4] Soundararajan M, Roos AK, Savitsky P, Filippakopoulos P, Kettenbach AN, Olsen JV, Gerber SA, Eswaran J, Knapp S, Elkins JM (2013). Structures of Down syndrome kinases, DYRKs, reveal mechanisms of kinase activation and substrate recognition. Structure.

[5] Himpel S, Tegge W, Frank R, Leder S, Joost H-G, Becker W (2000). Specificity determinants of substrate recognition by the protein kinase DYRK1A. J Biol Chem.

[6] Lochhead PA, Sibbet G, Morrice N, Cleghon V (2005). Activation-loop autophosphorylation is mediated by a novel transitional intermediate form of DYRKs. Cell.

[7] Cheng KC-C, Klancer R, Singson A, Seydoux G (2009). Regulation of MBK-2/DYRK by CDK-1 and the pseudophosphatases EGG-4 and EGG-5 during the oocyte-to-embryo transition. Cell.

[8] Parry JM, Velarde NV, Lefkovith AJ, Zegarek MH, Hang JS, Ohm J, Klancer R, Maruyama R, Druzhinina MK, Grant BD, Piano F, Singson A (2009). EGG-4 and EGG-5 link events of the oocyte-to-embryo transition with meiotic progression in C. elegans. Curr Biol.

[9] Han J, Miranda-Saavedra D, Luebbering N, Singh A, Sibbet G, Ferguson MAJ, Cleghon V (2012). Deep evolutionary conservation of an intramolecular protein kinase activation mechanism. PLoS ONE.

[10] Becker W, Weber Y, Wetzel K, Eirmbter K, Tejedor FJ, Joost H-G (1998). Sequence characteristics, subcellular localization, and substrate specificity of DYRK-related kinases, a novel family of dual specificity protein kinases. J Biol Chem.

[11] Lochhead PA, Sibbet G, Kinstrie R, Cleghon T, Rylatt M, Morrison DK, Cleghon V (2003). dDYRK2: a novel dual-specificity tyrosine-phosphorylation-regulated kinase in Drosophila. Biochem J.

[12] Houlgatte R, Mariage-Samson R, Duprat S, Tessier A, Bentolila S, Lamy B, Auffray C (1995). The Genexpress Index: a resource for gene discovery and the genic map of the human genome. Genome Res.

[13] Banerjee S, Ji C, Mayfield JE, Goel A, Xiao J, Dixon JE, Guo X (2018). Ancient drug curcumin impedes 26S proteasome activity by direct inhibition of dual-specificity tyrosine-regulated kinase 2. Proc Natl Acad Sci USA.

[14] Banerjee S, Wei T, Wang J, Lee JJ, Gutierrez HL, Chapman O, Wiley SE, Mayfield JE, Tandon V, Juarez EF, Chavez L, Liang R, Sah RL, Costello C, Mesirov JP, de la Vega L, Cooper KL, Dixon JE, Xiao J, Lei X (2019). Inhibition of dual-specificity tyrosine phosphorylation-regulated kinase 2 perturbs 26S proteasome-addicted neoplastic progression. Proc Natl Acad Sci USA.

[15] Kinstrie R, Luebbering N, Miranda-Saavedra D, Sibbet G, Han J, Lochhead PA, Cleghon V (2010). Characterization of a domain that transiently converts class 2 DYRKs into intramolecular tyrosine kinases. Sci Signal.

[16] Galceran J, de Graaf K, Tejedor FJ, Becker W (2003). The MNB/DYRK1A protein kinase: genetic and biochemical properties. J Neural Transm Suppl.

[17] Taira N, Yamamoto H, Yamaguchi T, Miki Y, Yoshida K (2010). ATM augments nuclear stabilization of DYRK2 by inhibiting MDM2 in the apoptotic response to DNA damage. J Biol Chem.

[18] Park CS, Lewis AH, Chen TJ, Bridges CS, Shen Y, Suppipat K, Puppi M, Tomolonis JA, Pang PD, Mistretta T-A, Ma L, Green MR, Rau R, Lacorazza HD (2019). A KLF4-DYRK2-mediated pathway regulating self-renewal in CML stem cells. Blood.

[19] Kumamoto T, Yamada K, Yoshida S, Aoki K, Hirooka S, Eto K, Yanaga K, Yoshida K (2020). Impairment of DYRK2 by DNMT1-mediated transcription augments carcinogenesis in human colorectal cancer. Int J Oncol.

[20] Kumamoto T, Yamada K, Yoshida S, Aoki K, Yanaga K, Yoshida K (2019). DNA methylation of dual-specificity tyrosine-regulated kinase 2 (DYRK2) promoter regulates proliferation of human colorectal cancer. J Am Coll Surg.

[21] Wang J, Jia Z, Zhang C, Sun M, Wang W, Chen P, Ma K, Zhang Y, Li X, Zhou C (2014). miR-499 protects cardiomyocytes from H 2O 2-induced apoptosis via its effects on Pdcd4 and Pacs2. RNA Biol.

[22] Wang Y, Sun J, Wei X, Luan L, Zeng X, Wang C, Zhao W (2017). Decrease of miR-622 expression suppresses migration and invasion by targeting regulation of DYRK2 in colorectal cancer cells. Onco Targets Ther.

[23] Yang J, Yu X, Xue F, Li Y, Liu W, Zhang S (2018). Exosomes derived from cardiomyocytes promote cardiac fibrosis via myocyte-fibroblast cross-talk. Am J Transl Res.

[24] Wang Y-J, Yue M, Guo K, Wu S-J, Tian Y-G (2018) The miR-338–3p involve in response to acute radiation syndrome by targeting DYRK2 in Tibet minipig. bioRxiv:506444. 10.1101/506444

[25] Haenisch S, von Ruden EL, Wahmkow H, Rettenbeck ML, Michler C, Russmann V, Bruckmueller H, Waetzig V, Cascorbi I, Potschka H (2016). miRNA-187-3p-mediated regulation of the KCNK10/TREK-2 potassium channel in a rat epilepsy model. ACS Chem Neurosci.

[26] Nolen B, Taylor S, Ghosh G (2004). Regulation of protein kinases; controlling activity through activation segment conformation. Mol Cell.

[27] Lim S, Jin K, Friedman E (2002). Mirk protein kinase is activated by MKK3 and functions as a transcriptional activator of HNF1alpha. J Biol Chem.

[28] Kii I, Sumida Y, Goto T, Sonamoto R, Okuno Y, Yoshida S, Kato-Sumida T, Koike Y, Abe M, Nonaka Y, Ikura T, Ito N, Shibuya H, Hosoya T, Hagiwara M (2016). Selective inhibition of the kinase DYRK1A by targeting its folding process. Nat Commun.

[29] Ashford AL, Dunkley TPJ, Cockerill M, Rowlinson RA, Baak LM, Gallo R, Balmanno K, Goodwin LM, Ward RA, Lochhead PA, Guichard S, Hudson K, Cook SJ (2016). Identification of DYRK1B as a substrate of ERK1/2 and characterisation of the kinase activity of DYRK1B mutants from cancer and metabolic syndrome. Cell Mol Life Sci.

[30] Kim K, Cha JS, Cho Y-S, Kim H, Chang N, Kim H-J, Cho H-S (2018). Crystal structure of human dual-specificity tyrosine-regulated kinase 3 reveals new structural features and insights into its auto-phosphorylation. J Mol Biol.

[31] Alvarez M, Altafaj X, Aranda S, de la Luna S (2007). DYRK1A autophosphorylation on serine residue 520 modulates its kinase activity via 14–3-3 binding. Mol Biol Cell.

[32] Taira N, Nihira K, Yamaguchi T, Miki Y, Yoshida K (2007). DYRK2 is targeted to the nucleus and controls p53 via Ser46 phosphorylation in the apoptotic response to DNA damage. Mol Cell.

[33] Pérez M, García-Limones C, Zapico I, Marina A, Schmitz ML, Muñoz E, Calzado MA (2012). Mutual regulation between SIAH2 and DYRK2 controls hypoxic and genotoxic signaling pathways. J Mol Cell Biol.

[34] Varjosalo M, Bjorklund M, Cheng F, Syvanen H, Kivioja T, Kilpinen S, Sun Z, Kallioniemi O, Stunnenberg HG, He WW, Ojala P, Taipale J (2008). Application of active and kinase-deficient kinome collection for identification of kinases regulating hedgehog signaling. Cell.

[35] Xu L, Sun Y, Li M, Ge X (2018). Dyrk2 mediated the release of proinflammatory cytokines in LPS-induced BV2 cells. Int J Biol Macromol.

[36] Hossain D, Javadi Esfehani Y, Das A, Tsang WY (2017). Cep78 controls centrosome homeostasis by inhibiting EDD-DYRK2-DDB1. EMBO Rep.

[37] Hornbeck PV, Zhang B, Murray B, Kornhauser JM, Latham V, Skrzypek E (2015). PhosphoSitePlus, 2014: mutations, PTMs and recalibrations. Nucleic Acids Res.

[38] Huang H, Arighi CN, Ross KE, Ren J, Li G, Chen SC, Wang Q, Cowart J, Vijay-Shanker K, Wu CH (2018). iPTMnet: an integrated resource for protein post-translational modification network discovery. Nucleic Acids Res.

[39] Campbell LE, Proud CG (2002). Differing substrate specificities of members of the DYRK family of arginine-directed protein kinases. FEBS Lett.

[40] Singh R, Lauth M (2017). Emerging roles of DYRK kinases in embryogenesis and hedgehog pathway control. J Dev Biol.

[41] Tanaka H, Morimura R, Ohshima T (2012). Dpysl2 (CRMP2) and Dpysl3 (CRMP4) phosphorylation by Cdk5 and DYRK2 is required for proper positioning of rohon-beard neurons and neural crest cells during neurulation in zebrafish. Dev Biol.

[42] Nishi Y, Lin R (2005). DYRK2 and GSK-3 phosphorylate and promote the timely degradation of OMA-1, a key regulator of the oocyte-to-embryo transition in C. elegans. Dev Biol.

[43] Mimoto R, Taira N, Takahashi H, Yamaguchi T, Okabe M, Uchida K, Miki Y, Yoshida K (2013). DYRK2 controls the epithelial-mesenchymal transition in breast cancer by degrading Snail. Cancer Lett.

[44] Taira N, Mimoto R, Kurata M, Yamaguchi T, Kitagawa M, Miki Y, Yoshida K (2012). DYRK2 priming phosphorylation of c-Jun and c-Myc modulates cell cycle progression in human cancer cells. J Clin Investig.

[45] Woods YL, Cohen P, Becker W, Jakes R, Goedert M, Wang X, Proud CG (2001). The kinase DYRK phosphorylates protein-synthesis initiation factor eIF2Bepsilon at Ser539 and the microtubule-associated protein tau at Thr212: potential role for DYRK as a glycogen synthase kinase 3-priming kinase. Biochem J.

[46] Cole AR, Causeret F, Yadirgi G, Hastie CJ, McLauchlan H, McManus EJ, Hernandez F, Eickholt BJ, Nikolic M, Sutherland C (2006). Distinct priming kinases contribute to differential regulation of collapsin response mediator proteins by glycogen synthase kinase-3 in vivo. J Biol Chem.

[47] Beurel E, Grieco SF, Jope RS (2015). Glycogen synthase kinase-3 (GSK3): regulation, actions, and diseases. Pharmacol Ther.

[48] Jope RS, Johnson GV (2004). The glamour and gloom of glycogen synthase kinase-3. Trends Biochem Sci.

[49] Doble BW, Woodgett JR (2003). GSK-3: tricks of the trade for a multi-tasking kinase. J Cell Sci.

[50] Thomas GM, Frame S, Goedert M, Nathke I, Polakis P, Cohen P (1999). A GSK3-binding peptide from FRAT1 selectively inhibits the GSK3-catalysed phosphorylation of axin and beta-catenin. FEBS Lett.

[51] Maddika S, Chen J (2009). Protein kinase DYRK2 is a scaffold that facilitates assembly of an E3 ligase. Nat Cell Biol.

[52] Jung H-Y, Wang X, Jun S, Park J-I (2013). Dyrk2-associated EDD-DDB1-VprBP E3 ligase inhibits telomerase by TERT degradation. J Biol Chem.

[53] Chatterjee N, Walker GC (2017). Mechanisms of DNA damage, repair, and mutagenesis. Environ Mol Mutagen.

[54] Yogosawa S, Yoshida K (2018). Tumor suppressive role for kinases phosphorylating p53 in DNA damage-induced apoptosis. Cancer Sci.

[55] Yamamoto T, Taira Nihira N, Yogosawa S, Aoki K, Takeda H, Sawasaki T, Yoshida K (2017). Interaction between RNF8 and DYRK2 is required for the recruitment of DNA repair molecules to DNA double-strand breaks. FEBS Lett.

[56] Luebbering N, Charlton-Perkins M, Kumar JP, Rollmann SM, Cook T, Cleghon V (2013). Drosophila Dyrk2 plays a role in the development of the visual system. PLoS ONE.

[57] Raich WB, Moorman C, Lacefield CO, Lehrer J, Bartsch D, Plasterk RHA, Kandel ER, Hobert O (2003). Characterization of Caenorhabditis elegans homologs of the down syndrome candidate gene DYRK1A. Genetics.

[58] Sun W, Jiao S, Tan X, Zhang P, You F (2017). DYRK2 displays muscle fiber type specific function during zebrafish early somitogenesis. Int J Dev Biol.

[59] Woo Y, Kim SJ, Suh BK (2019). Sequential phosphorylation of NDEL1 by the DYRK2-GSK3beta complex is critical for neuronal morphogenesis. Elife.

[60] Cayla M, McDonald L, MacGregor P, Matthews K (2020). An atypical DYRK kinase connects quorum-sensing with posttranscriptional gene regulation in Trypanosoma brucei. Elife.

[61] Gwack Y, Sharma S, Nardone J, Tanasa B, Iuga A, Srikanth S, Okamura H, Bolton D, Feske S, Hogan PG, Rao A (2006). A genome-wide Drosophila RNAi screen identifies DYRK-family kinases as regulators of NFAT. Nature.

[62] Morrugares R, Correa-Sáez A, Moreno R, Garrido-Rodríguez M, Muñoz E, de la Vega L, Calzado MA (2019). Phosphorylation-dependent regulation of the NOTCH1 intracellular domain by dual-specificity tyrosine-regulated kinase 2. Cell Mol Life Sci.

[63] An T, Li S, Pan W, Tien P, Zhong B, Shu HB, Wu S (2015). DYRK2 negatively regulates type I interferon induction by promoting TBK1 degradation via Ser527 phosphorylation. PLoS Pathog.

[64] Moreno R, Banerjee S, Jackson AW, Quinn J, Baillie G, Dixon JE, Dinkova-Kostova AT, Edwards J, de la Vega L (2019) DYRK2 activates heat shock factor 1 promoting resistance to proteotoxic stress in triplenegative breast cancer. bioRxiv 10.1038/s41418-020-00686-8 PMC8166837 33268814

[65] Imawari Y, Mimoto R, Hirooka S, Morikawa T, Takeyama H, Yoshida K (2018). Downregulation of dual-specificity tyrosine-regulated kinase 2 promotes tumor cell proliferation and invasion by enhancing cyclin-dependent kinase 14 expression in breast cancer. Cancer Sci.

[66] Guo X, Wang X, Wang Z, Banerjee S, Yang J, Huang L, Dixon JE (2016). Site-specific proteasome phosphorylation controls cell proliferation and tumorigenesis. Nat Cell Biol.

[67] Ong SS, Goktug AN, Elias A, Wu J, Saunders D, Chen T (2014). Stability of the human pregnane X receptor is regulated by E3 ligase UBR5 and serine/threonine kinase DYRK2. Biochem J.

[68] Weiss CS, Ochs MM, Hagenmueller M, Streit MR, Malekar P, Riffel JH, Buss SJ, Weiss KH, Sadoshima J, Katus HA, Hardt SE (2013). DYRK2 negatively regulates cardiomyocyte growth by mediating repressor function of GSK-3beta on eIF2Bepsilon. PLoS ONE.

[69] Miller CT, Aggarwal S, Lin TK, Dagenais SL, Contreras JI, Orringer MB, Glover TW, Beer DG, Lin L (2003). Amplification and overexpression of the dual-specificity tyrosine-(Y)-phosphorylation regulated kinase 2 (DYRK2) gene in esophageal and lung adenocarcinomas. Can Res.

[70] Shen Y, Zhang L, Wang D, Bao Y, Liu C, Xu Z, Huang W, Cheng C (2017). Regulation of glioma cells migration by DYRK2. Neurochem Res.

[71] Becker W (2012). Emerging role of DYRK family protein kinases as regulators of protein stability in cell cycle control. Cell Cycle (Georgetown, Tex).

[72] Zhou W, Lv R, Qi W, Wu D, Xu Y, Liu W, Mou Y, Wang L (2014). Snail contributes to the maintenance of stem cell-like phenotype cells in human pancreatic cancer. PLoS ONE.

[73] Ryu K-J, Park S-M, Park S-H, Kim I-K, Han H-T, Kim H-J, Kim SH, Hong K-S, Kim H, Kim M, Yoon S-J, Asaithambi K, Lee KH, Park J-Y, Hah Y-S, Cho HJ, Yook JI, Yang JW, Ko G-H, Lee G, Kang YJ, Hwangbo C, Kim KD, Park Y-J, Yoo J (2019). p38 stabilizes snail by suppressing DYRK2-mediated phosphorylation that is required for GSK3β-βTrCP-induced snail degradation. Cancer Res.

[74] Yamaguchi N, Mimoto R, Yanaihara N, Imawari Y, Hirooka S, Okamoto A, Yoshida K (2015). DYRK2 regulates epithelial-mesenchymal-transition and chemosensitivity through Snail degradation in ovarian serous adenocarcinoma. Tumour Biol.

[75] Mrugala MM (2013). Advances and challenges in the treatment of glioblastoma: a clinician's perspective. Discov Med.

[76] Ito D, Yogosawa S, Mimoto R, Hirooka S, Horiuchi T, Eto K, Yanaga K, Yoshida K (2017). Dual-specificity tyrosine-regulated kinase 2 is a suppressor and potential prognostic marker for liver metastasis of colorectal cancer. Cancer Sci.

[77] Yan H, Hu K, Wu W, Li Y, Tian H, Chu Z, Koeffler HP, Yin D (2016). Low expression of DYRK2 (dual specificity tyrosine phosphorylation regulated kinase 2) correlates with poor prognosis in colorectal cancer. PLoS ONE.

[78] Mimoto R, Imawari Y, Hirooka S, Takeyama H, Yoshida K (2017). Impairment of DYRK2 augments stem-like traits by promoting KLF4 expression in breast cancer. Oncogene.

[79] Yoshida S, Yoshida K (2019). Multiple functions of DYRK2 in cancer and tissue development. FEBS Lett.

[80] Zhang X, Xu P, Ni W, Fan H, Xu J, Chen Y, Huang W, Lu S, Liang L, Liu J, Chen B, Shi W (2016). Downregulated DYRK2 expression is associated with poor prognosis and oxaliplatin resistance in hepatocellular carcinoma. Pathol Res Pract.

[81] Yamashita S, Chujo M, Moroga T, Anami K, Tokuishi K, Miyawaki M, Kawano Y, Takeno S, Yamamoto S, Kawahara K (2009). DYRK2 expression may be a predictive marker for chemotherapy in non-small cell lung cancer. Anticancer Res.

[82] Yamashita S, Chujo M, Tokuishi K, Anami K, Miyawaki M, Yamamoto S, Kawahara K (2009). Expression of dual-specificity tyrosine-(Y)-phosphorylation-regulated kinase 2 (DYRK2) can be a favorable prognostic marker in pulmonary adenocarcinoma. J Thorac Cardiovasc Surg.

[83] Nomura S, Suzuki Y, Takahashi R, Terasaki M, Kimata R, Terasaki Y, Hamasaki T, Kimura G, Shimizu A, Kondo Y (2015). Dual-specificity tyrosine phosphorylation-regulated kinase 2 (DYRK2) as a novel marker in T1 high-grade and T2 bladder cancer patients receiving neoadjuvant chemotherapy. BMC Urol.

[84] Wang Y, Wu Y, Miao X, Zhu X, Miao X, He Y, Zhong F, Ding L, Liu J, Tang J, Huang Y, Xu X, He S (2015). Silencing of DYRK2 increases cell proliferation but reverses CAM-DR in Non-Hodgkin's Lymphoma. Int J Biol Macromol.

[85] Uhl KL, Schultz CR, Geerts D, Bachmann AS (2018). Harmine, a dual-specificity tyrosine phosphorylation-regulated kinase (DYRK) inhibitor induces caspase-mediated apoptosis in neuroblastoma. Cancer Cell Int.

[86] Enomoto Y, Yamashita S, Yoshinaga Y, Fukami Y, Miyahara S, Nabeshima K, Iwasaki A (2014). Downregulation of DYRK2 can be a predictor of recurrence in early stage breast cancer. Tumour Biol.

[87] Sun Y, Ge X, Li M, Xu L, Shen Y (2017). Dyrk2 involved in regulating LPS-induced neuronal apoptosis. Int J Biol Macromol.

[88] Moloudizargari M, Mikaili P, Aghajanshakeri S, Asghari MH, Shayegh J (2013). Pharmacological and therapeutic effects of Peganum harmala and its main alkaloids. Pharmacogn Rev.

[89] Zhang L, Zhang F, Zhang W, Chen L, Gao N, Men Y, Xu X, Jiang Y (2015). Harmine suppresses homologous recombination repair and inhibits proliferation of hepatoma cells. Cancer Biol Ther.

[90] Gockler N, Jofre G, Papadopoulos C, Soppa U, Tejedor FJ, Becker W (2009). Harmine specifically inhibits protein kinase DYRK1A and interferes with neurite formation. FEBS J.

[91] Ogawa Y, Nonaka Y, Goto T, Ohnishi E, Hiramatsu T, Kii I, Yoshida M, Ikura T, Onogi H, Shibuya H, Hosoya T, Ito N, Hagiwara M (2010). Development of a novel selective inhibitor of the down syndrome-related kinase Dyrk1A. Nat Commun.

[92] Becker W, Soppa U, Tejedor FJ (2014). DYRK1A: a potential drug target for multiple down syndrome neuropathologies. CNS Neurol Disord Drug Targets.

[93] Matthay KK, Maris JM, Schleiermacher G, Nakagawara A, Mackall CL, Diller L, Weiss WA (2016). Neuroblastoma. Nat Rev Dis Primers.

[94] Nelson KM, Dahlin JL, Bisson J, Graham J, Pauli GF, Walters MA (2017). The essential medicinal chemistry of curcumin. J Med Chem.

[95] Cuny GD, Robin M, Ulyanova NP, Patnaik D, Pique V, Casano G, Liu JF, Lin X, Xian J, Glicksman MA, Stein RL, Higgins JM (2010). Structure-activity relationship study of acridine analogs as haspin and DYRK2 kinase inhibitors. Bioorg Med Chem Lett.

[96] Cuny GD, Ulyanova NP, Patnaik D, Liu JF, Lin X, Auerbach K, Ray SS, Xian J, Glicksman MA, Stein RL, Higgins JM (2012). Structure-activity relationship study of beta-carboline derivatives as haspin kinase inhibitors. Bioorg Med Chem Lett.

[97] Miyabayashi T, Yamamoto M, Sato A, Sakano S, Takahashi Y (2008). Indole derivatives sustain embryonic stem cell self-renewal in long-term culture. Biosci Biotechnol Biochem.

[98] Hasegawa K, Yasuda SY, Teo JL, Nguyen C, McMillan M, Hsieh CL, Suemori H, Nakatsuji N, Yamamoto M, Miyabayashi T, Lutzko C, Pera MF, Kahn M (2012). Wnt signaling orchestration with a small molecule DYRK inhibitor provides long-term xeno-free human pluripotent cell expansion. Stem Cells Transl Med.

[99] Ribas J, Bettayeb K, Ferandin Y, Knockaert M, Garrofe-Ochoa X, Totzke F, Schachtele C, Mester J, Polychronopoulos P, Magiatis P, Skaltsounis AL, Boix J, Meijer L (2006). 7-Bromoindirubin-3'-oxime induces caspase-independent cell death. Oncogene.

[100] Myrianthopoulos V, Kritsanida M, Gaboriaud-Kolar N, Magiatis P, Ferandin Y, Durieu E, Lozach O, Cappel D, Soundararajan M, Filippakopoulos P, Sherman W, Knapp S, Meijer L, Mikros E, Skaltsounis AL (2013). Novel inverse binding mode of indirubin derivatives yields improved selectivity for DYRK kinases. ACS Med Chem Lett.

[101] Myrianthopoulos V, Magiatis P, Ferandin Y, Skaltsounis AL, Meijer L, Mikros E (2007). An integrated computational approach to the phenomenon of potent and selective inhibition of aurora kinases B and C by a series of 7-substituted indirubins. J Med Chem.

[102] Ashford AL, Oxley D, Kettle J, Hudson K, Guichard S, Cook SJ, Lochhead PA (2014). A novel DYRK1B inhibitor AZ191 demonstrates that DYRK1B acts independently of GSK3beta to phosphorylate cyclin D1 at Thr(286), not Thr(288). Biochem J.

[103] Uhlen M, Fagerberg L, Hallstrom BM, Lindskog C, Oksvold P, Mardinoglu A, Sivertsson A, Kampf C, Sjostedt E, Asplund A, Olsson I, Edlund K, Lundberg E, Navani S, Szigyarto CA, Odeberg J, Djureinovic D, Takanen JO, Hober S, Alm T, Edqvist PH, Berling H, Tegel H, Mulder J, Rockberg J, Nilsson P, Schwenk JM, Hamsten M, von Feilitzen K, Forsberg M, Persson L, Johansson F, Zwahlen M, von Heijne G, Nielsen J, Ponten F (2015). Proteomics. Tissue-based map of the human proteome. Science.

[104] Stark C, Breitkreutz BJ, Reguly T, Boucher L, Breitkreutz A, Tyers M (2006). BioGRID: a general repository for interaction datasets. Nucleic Acids Res.

[105] Liberzon A, Birger C, Thorvaldsdottir H, Ghandi M, Mesirov JP, Tamayo P (2015). The molecular signatures database (MSigDB) hallmark gene set collection. Cell Syst.

[106] Yu G, Wang LG, Han Y, He QY (2012). clusterProfiler: an R package for comparing biological themes among gene clusters. OMICS.

[107] Deng M, Bragelmann J, Kryukov I, Saraiva-Agostinho N, Perner S (2017). FirebrowseR: an R client to the Broad Institute's firehose pipeline. Database (Oxford).

[108] Barretina J, Caponigro G, Stransky N, Venkatesan K, Margolin AA, Kim S, Wilson CJ, Lehar J, Kryukov GV, Sonkin D, Reddy A, Liu M, Murray L, Berger MF, Monahan JE, Morais P, Meltzer J, Korejwa A, Jane-Valbuena J, Mapa FA, Thibault J, Bric-Furlong E, Raman P, Shipway A, Engels IH, Cheng J, Yu GK, Yu J, Aspesi P, de Silva M, Jagtap K, Jones MD, Wang L, Hatton C, Palescandolo E, Gupta S, Mahan S, Sougnez C, Onofrio RC, Liefeld T, MacConaill L, Winckler W, Reich M, Li N, Mesirov JP, Gabriel SB, Getz G, Ardlie K, Chan V, Myer VE, Weber BL, Porter J, Warmuth M, Finan P, Harris JL, Meyerson M, Golub TR, Morrissey MP, Sellers WR, Schlegel R, Garraway LA (2012). The cancer cell line encyclopedia enables predictive modelling of anticancer drug sensitivity. Nature.

[109] Cerami E, Gao J, Dogrusoz U, Gross BE, Sumer SO, Aksoy BA, Jacobsen A, Byrne CJ, Heuer ML, Larsson E, Antipin Y, Reva B, Goldberg AP, Sander C, Schultz N (2012). The cBio cancer genomics portal: an open platform for exploring multidimensional cancer genomics data. Cancer Discov.

[110] Auld GC, Campbell DG, Morrice N, Cohen P (2005). Identification of calcium-regulated heat-stable protein of 24 kDa (CRHSP24) as a physiological substrate for PKB and RSK using KESTREL. Biochem J.

[111] Cole AR, Knebel A, Morrice NA, Robertson LA, Irving AJ, Connolly CN, Sutherland C (2004). GSK-3 phosphorylation of the Alzheimer epitope within collapsin response mediator proteins regulates axon elongation in primary neurons. J Biol Chem.

[112] Slepak TI, Salay LD, Lemmon VP, Bixby JL (2012). Dyrk kinases regulate phosphorylation of doublecortin, cytoskeletal organization, and neuronal morphology. Cytoskeleton (Hoboken).

[113] Wang X, Li W, Parra JL, Beugnet A, Proud CG (2003). The C terminus of initiation factor 4E-binding protein 1 contains multiple regulatory features that influence its function and phosphorylation. Mol Cell Biol.

[114] Skurat AV, Dietrich AD (2004). Phosphorylation of Ser640 in muscle glycogen synthase by DYRK family protein kinases. J Biol Chem.

[115] Tsai CF, Wang YT, Yen HY, Tsou CC, Ku WC, Lin PY, Chen HY, Nesvizhskii AI, Ishihama Y, Chen YJ (2015). Large-scale determination of absolute phosphorylation stoichiometries in human cells by motif-targeting quantitative proteomics. Nat Commun.

[116] Matsuo R, Ochiai W, Nakashima K, Taga T (2001). A new expression cloning strategy for isolation of substrate-specific kinases by using phosphorylation site-specific antibody. J Immunol Methods.

[117] Huttlin EL, Jedrychowski MP, Elias JE, Goswami T, Rad R, Beausoleil SA, Villen J, Haas W, Sowa ME, Gygi SP (2010). A tissue-specific atlas of mouse protein phosphorylation and expression. Cell.

[118] Yokoyama-Mashima S, Yogosawa S, Kanegae Y, Hirooka S, Yoshida S, Horiuchi T, Ohashi T, Yanaga K, Saruta M, Oikawa T, Yoshida K (2019). Forced expression of DYRK2 exerts anti-tumor effects via apoptotic induction in liver cancer. Cancer Lett.

[119] Moreno P, Lara-Chica M, Soler-Torronteras R, Caro T, Medina M, Álvarez A, Salvatierra Á, Munoz E, Calzado Canale MA (2015). The expression of the ubiquitin ligase SIAH2 (seven in Absentia Homolog 2) is increased in human lung cancer. PLoS ONE.

