# Supplementary figures and images for: Updating dual-specificity tyrosine-phosphorylation-regulated kinase 2 (DYRK2): molecular basis, functions and role in diseases
Source: Cell Mol Life Sci. 2020 May 27;77(23):4747–63. doi: 10.1007/s00018-020-03556-1 (PMC7658070; doi:10.1007/s00018-020-03556-1)

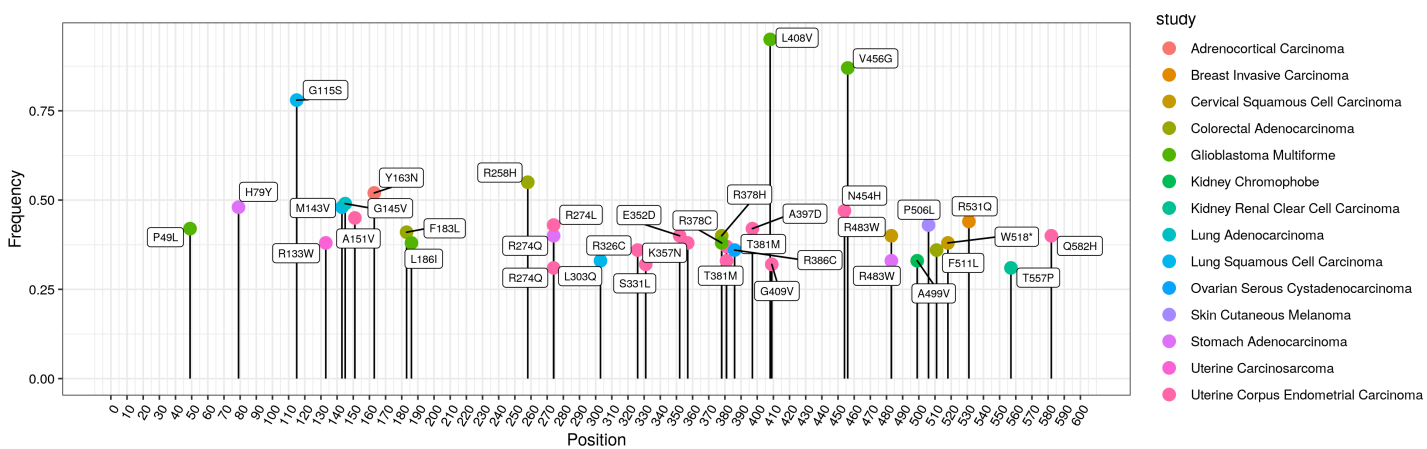

Supplement: Supplementary file 1 — Supplementary file1 (PDF 613 kb) [file 18_2020_3556_MOESM1_ESM.pdf]
