# Supplementary material for: Updating dual-specificity tyrosine-phosphorylation-regulated kinase 2 (DYRK2): molecular basis, functions and role in diseases
Source: Cell Mol Life Sci. 2020 May 27;77(23):4747–63. doi: 10.1007/s00018-020-03556-1 (PMC7658070; doi:10.1007/s00018-020-03556-1)
Supplement: Supplementary file 2 — Supplementary file2 (PDF 97 kb) [file 18_2020_3556_MOESM2_ESM.pdf]

Supplementary Material for:

Updating dual specificity tyrosine-phosphorylation-regulated kinase 2 (DYRK2): molecular basis, functions and role in diseases.

Alejandro Correa-Sáez[1,2,3], Rafael Jiménez-Izquierdo[1,2,3], Martín Garrido-Rodríguez[1,2,3], Rosario Morrugares[1,2,3], Eduardo Muñoz[1,2,3] and Marco A. Calzado[1,2,3]

[1] Instituto Maimónides de Investigación Biomédica de Córdoba (IMIBIC), Córdoba, Spain.

[2] Departamento de Biología Celular, Fisiología e Inmunología, Universidad de Córdoba, Córdoba, Spain.

[3] Hospital Universitario Reina Sofía, Córdoba, Spain.

Corresponding Author: Marco A. Calzado Ph.D.

Instituto Maimónides de Investigación Biomédica de Córdoba (IMIBIC)

Avda. Menéndez Pidal s/n. 14004, Córdoba, Spain

Phone: + 34 957213762

e-mail: mcalzado@uco.es

Supplementary figures.

Supplementary Figure S1. DYRK2 mutations in cancer. The DYRK2 mutation list for the TCGA PanCancer Atlas Studies was obtained from cBioPortal (https://www.cbioportal.org/)[109] and filtered to variants with a tumor allele frequency > 0.3. For each mutation (point), the color indicates the TCGA study where it was detected, the X axis the position on DYRK2 protein and the Y axis the tumor allele frequency.

Supplementary Table

Site	Organism	PTM type	Sources
**S7**	Homo sapiens (human)	Phosphorylation	PhosphoSitePlus
**R18**	Homo sapiens (human)	Methylation	PhosphoSitePlus
**S30**	Homo sapiens (human)	Phosphorylation	PhosphoSitePlus, iPTMNet
**S30**	Mus musculus (mouse)	Phosphorylation	PhosphoSitePlus
**T44**	Homo sapiens (human)	Phosphorylation	PhosphoSitePlus
**S48**	Homo sapiens (human)	Phosphorylation	PhosphoSitePlus
**S48**	Mus musculus (mouse)	Phosphorylation	PhosphoSitePlus
**T106**	Homo sapiens (human)	Phosphorylation	PhosphoSitePlus, iPTMNet
**S142**	Homo sapiens (human)	Phosphorylation	PhosphoSitePlus
**Y308**	Rattus norvegicus (rat)	Phosphorylation	PhosphoSitePlus
**Y314**	Rattus norvegicus (rat)	Phosphorylation	PhosphoSitePlus
**C347**	Homo sapiens (human)	S-Nitrosylation	iPTMNet
**C373**	Homo sapiens (human)	S-Nitrosylation	iPTMNet
**T379**	Mus musculus (mouse)	Phosphorylation	PhosphoSitePlus
**Y380**	Homo sapiens (human)	Phosphorylation	PhosphoSitePlus, iPTMNet
**Y380**	Mus musculus (mouse)	Phosphorylation	PhosphoSitePlus
**T381**	Homo sapiens (human)	Phosphorylation	PhosphoSitePlus, iPTMNet
**Y382**	Homo sapiens (human)	Phosphorylation	PhosphoSitePlus, iPTMNet
**S385**	Homo sapiens (human)	Phosphorylation	PhosphoSitePlus
**S442**	Homo sapiens (human)	Phosphorylation	PhosphoSitePlus, iPTMNet
**S449**	Homo sapiens (human)	Phosphorylation	PhosphoSitePlus, iPTMNet
**Y464**	Homo sapiens (human)	Phosphorylation	PhosphoSitePlus, iPTMNet
**T525**	Homo sapiens (human)	Phosphorylation	PhosphoSitePlus, iPTMNet

Supplementary Table 1. DYRK2 post-translational modifications extracted from the databases PhosphoSitePlus (https://www.phosphosite.org/homeAction.action) and iPTMNet (https://research.bioinformatics.udel.edu/iptmnet/).
